# Financial Burden and Its Impact on the Lives of Family Caregivers With an Older Long-Term Care Recipient

**DOI:** 10.1093/geroni/igaf122.2332

**Published:** 2025-12-31

**Authors:** Ayumi Honda

**Affiliations:** St. Mary’s College, Kurume, Fukuoka, Japan

## Abstract

This study used a cross-sectional design to examine caregiver characteristics and financial burden associated with the cost of utilizing long-term care services for an older relative among 427 family caregivers. The proportion of family caregivers experiencing financial burden was significantly higher among those living with an older care recipient compared to those whose older care recipient was in a care facility (68.2% vs. 57.3%; p = 0.049). Among 198 family caregivers living with an older care recipient, greater severity of functional disability in the recipient was significantly associated with financial burden and its impact on their lives (p = 0.004). On the other hand, among 229 family caregivers of an older care recipient utilizing institutional care, those bearing the costs of long-term care were more likely to experience financial burden and its impact on their lives (p < 0.001). Among the family caregivers, experiencing financial burden and its impact on their lives related to poorer health and greater caregiver burden, along with a lower annual household income and bearing the caregiving costs. We found that financial shortages in older care recipients had a negative impact on their family caregivers’ work, private lives, and care choices. Community-based family care plays a crucial role in supporting stress coping for family caregivers facing financial hardship.

